# Phosphoglyceric acid mutase-1 contributes to oncogenic mTOR-mediated tumor growth and confers non-small cell lung cancer patients with poor prognosis

**DOI:** 10.1038/s41418-017-0034-y

**Published:** 2018-01-23

**Authors:** Qian Sun, Shuzhan Li, Yanan Wang, Haiyong Peng, Xiying Zhang, Yu Zheng, Chunjia Li, Li Li, Rongrong Chen, Xinxin Chen, Wenjing Bai, Xiangli Jiang, Liang Liu, Feng Wei, Boshi Wang, Yu Zhang, Hui Li, Xiubao Ren, Hongbing Zhang

**Affiliations:** 1 Department of Immunology, Key Laboratory of Cancer Immunology and Biotherapy, Key Laboratory of Cancer Prevention and Therapy, National Clinical Research Center of Cancer, Tianjin’s Clinical Research Center for Cancer, Tianjin Medical University Cancer Institute and Hospital, Tianjin, 300060 China; 2 State Key Laboratory of Medical Molecular Biology, Department of Physiology, Institute of Basic Medical Sciences, Chinese Academy of Medical Sciences and School of Basic Medicine, Peking Union Medical College, Beijing, 100005 China; 3 Department of Thoracic Medical Oncology, National Clinical Research Center of Cancer, Tianjin Medical University Cancer Institute and Hospital, Tianjin, 300060 China; 4 State Key laboratory of Oncogenes and Related Genes, Shanghai Cancer Institute, Renji Hospital, Shanghai Jiaotong University School of Medicine, Shanghai, 200032 China; 5 State Key Laboratory of Molecular Oncology, Cancer Institute (Hospital), Peking Union Medical College and Chinese Academy of Medical Sciences, Beijing, 100021 China

## Abstract

As a hallmark of cancer, the Warburg effect (aerobic glycolysis) confers a selective advantage for the survival and proliferation of cancer cells. Due to frequent aberration of upstream proto-oncogenes and tumor suppressors, hyperactive mammalian/mechanistic target of rapamycin (mTOR) is a potent inducer of the Warburg effect. Here, we report that overexpression of a glycolytic enzyme, phosphoglyceric acid mutase-1 (PGAM1), is critical to oncogenic mTOR-mediated Warburg effect. mTOR stimulated PGAM1 expression through hypoxia-inducible factor 1α-mediated transcriptional activation. Blockage of PGAM1 suppressed mTOR-dependent glycolysis, cell proliferation, and tumorigenesis. PGAM1 expression and mTOR activity were positively correlated in non-small cell lung cancer (NSCLC) tissues and PGAM1 abundance was an adverse predictor for patient survival. PGAM1 is thus a downstream effector of mTOR signaling pathway and mTOR-PGAM1 signaling cascade may contribute to the development of Warburg effect observed in cancer. We consider PGAM1 as a novel prognostic biomarker for NSCLC and a therapeutic target for cancer.

## Introduction

Normal cells metabolize glucose through tricarboxylic acid cycle (TCA cycle) under normoxic condition and glycolytic pathway under hypoxia condition. However, cancer cells primarily utilize glycolysis to consume glucose and produce lactate even in the presence of oxygen, this metabolic shift is termed as aerobic glycolysis [1–3]. This hallmark of cancer cells was first described by Otto Warburg and is thus also called the Warburg effect [4]. This inefficient energy production metabolism somehow renders a selective advantage for the survival and proliferation of cancer cells [1–3]. Therefore, mechanistic insights into the induction of Warburg effect and clinical relevance of this unique cancer metabolism were under intensive investigation in recent years.

The receptor tyrosine kinase (RTK)-phosphatidylinositol 3-kinase (PI3K)-AKT-mammalian/mechanistic target of rapamycin (mTOR) pathway plays important roles in the regulation of cell metabolism, survival, and proliferation [5, 6]. Genetic and epigenetic alterations of both proto-oncogenes and tumor suppressor genes in the upstream of mTOR bestow it one of the most frequently deregulated signaling pathways in human diseases, especially in cancer [7]. Serine/threonine protein kinase mTOR integrates the cues of nutrients and growth factors to regulate cell metabolism and growth [8–10]. We have found that mTOR promotes Warburg effect largely through up-regulation of several glycolytic enzymes including a rate-limiting enzyme, PKM2 [11, 12]. PKM2 is an embryonic M2 isoform of the glycolytic enzyme pyruvate kinase, which is critical for the regulation of cell metabolism and mainly expresses in proliferating cells, especially cancer cells [13, 14].

Phosphoglyceric acid mutase (PGAM) catalyzes the conversion of 3-phosphoglycerate (3-PG) into 2-phosphoglycerate (2-PG) in the late stage of glycolysis [15, 16]. There are two tissue-specific isoforms of PGAM: brain isoform (PGAM1) and muscle isoform (PGAM2) in human [17, 18]. PKM2 enhances phosphoenolpyruvate-dependent histidine phosphorylation of PGAM1 and the activated PGAM1 drives forward glycolysis [19]. On the other hand, Sirt1 suppressed glycolysis by deacetylating PGAM1 [20]. These post-translational modifications may rapidly modulate PGAM1 function [21]. PGAM1 exerts its dual catabolic and anabolic roles by coupling glycolysis with biosynthesis [21].

Elevated PGAM1 was observed in breast, lung, liver, colon, kidney, and urothelial bladder cancers [22–26]. TP53 is the most frequently mutated tumor suppressor gene. Conflicting results have been reported on p53 regulation of PGAM2 abundance [27, 28]. The signal transduction pathways that regulate PGAM1 expression were largely unknown. Since mTOR is a positive regulator of the Warburg effect and PGAM1 plays important roles in glycolysis and biosynthesis, we speculated that PGAM1 participated in mTOR-mediated glycolysis and oncogenesis.

In this study, we first investigated mTOR regulation of PGAM1 expression and then the role of PGAM1 in mTOR-mediated glycolysis and tumor growth. To elucidate the potential existence of mTOR-PGAM1 cascade in human cancers and its clinical relevance, we examined the relationship of mTOR activity and PGAM1 expression in human non-small cell lung cancer (NSCLC) samples.

## Results

### mTOR enhances PGAM1 expression

**Fig. 1:**
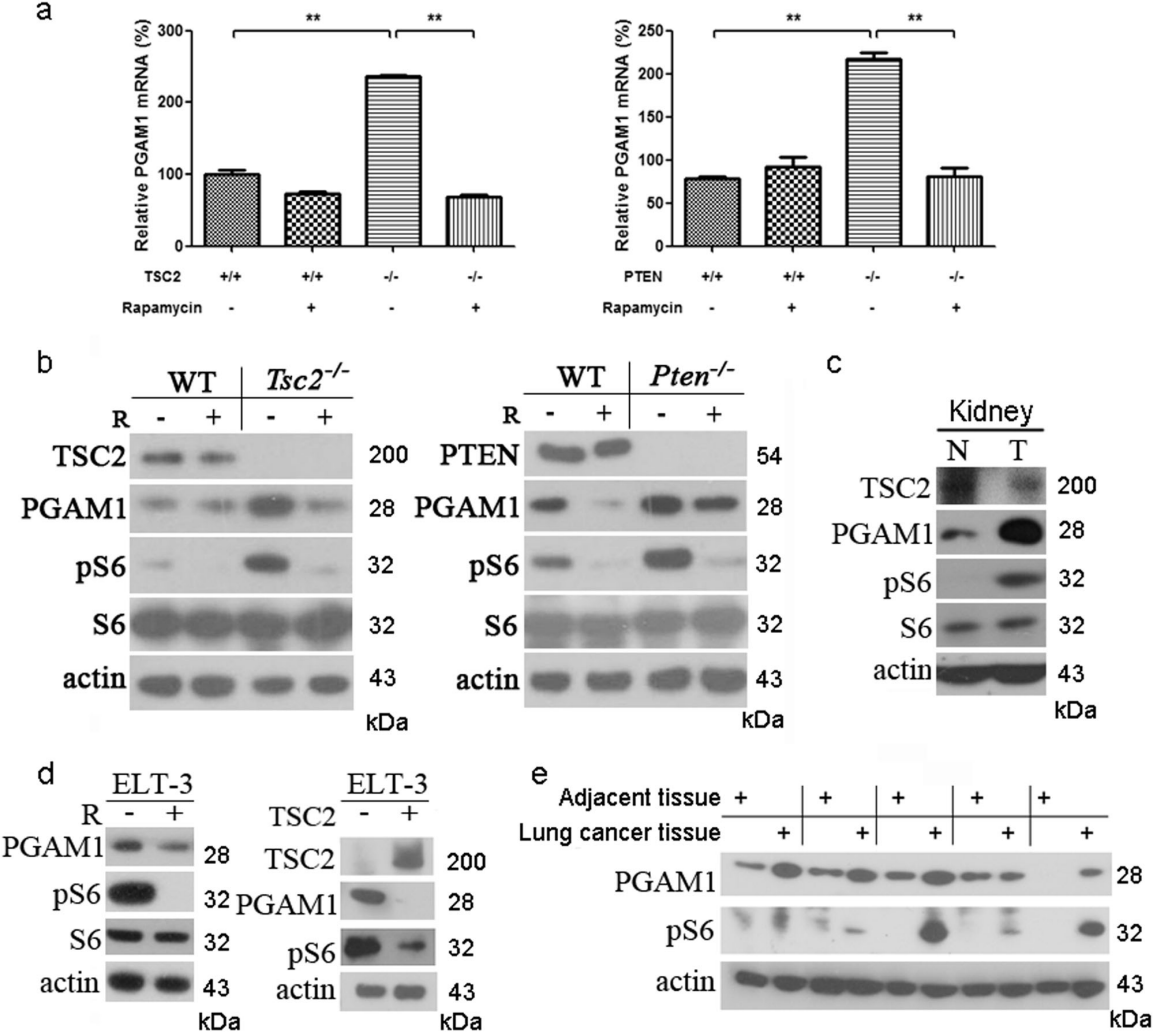
Activated mTOR stimulates PGAM1 expression. qRT-PCR **a** and immunoblotting **b** of cell lysates extracted from WT, *Tsc2*^*−/−*^, or *Pten*^*−/−*^ MEFs treated with or without 10 nM rapamycin (R) for 24 h. **c** Immunoblotting of lysates from kidney tumor and non-tumor kidney tissues of *Tsc2*^*del3/+*^ mouse. **d** Left panel: Rat ELT3 cells treated with or without 10 nM rapamycin for 24 h were subjected to immunoblotting. Right panel: Immunoblotting of ELT3 cells with/without restoration of TSC2. **e** Protein lysates were extracted from human NSCLC tissues and adjacent tissues and then subjected to immunoblotting. Phosphorylation of S6 (pS6) is a marker of mTOR activity

Tuberous sclerosis complex 1 (TSC1), TSC2, and PTEN (phosphatase and tensin homolog) tumor suppressors are major negative regulators of mTOR signaling pathway [29-33]. Loss of *TSC1*, *TSC2*, or *PTEN* leads to hyperactivation of mTOR and therefore the cells and tissues deficient of these tumor suppressors are widely used in the study of mTOR signaling [11,34–36]. Our previous work demonstrated that hyperactivation of mTOR signaling induced the Warburg effect in *Tsc1*, *Tsc2*, or *Pten* knockout mouse embryonic fibroblasts (MEFs), through up-regulation of a glycolytic enzyme, PKM2 [11]. Since glycolytic pathway is a 10-step reaction process involving several catalytic enzymes, we wondered whether other enzymes might participate in this metabolic aberration in cancer cells. To investigate the role of PGAM1 in mTOR-mediated tumorigenesis, we examined the mRNA and protein levels of PGAM1 in *Tsc2*^−/−^ and *Pten*^−/−^ MEFs compared with their wild-type counterparts. Phosphorylation level of ribosomal protein S6 (pS6) was used here as a readout of mTOR activation [34, 35]. Both PGAM1 mRNA and protein were increased in cells lacking *Tsc2* or *Pten*, and were decreased with treatment of mTOR inhibitor, rapamycin (Figs. 1a, b). PGAM1 was elevated in mouse kidney tumor caused by up-regulated mTOR due to *Tsc2* exon 3 deletion (*Tsc2*^del3/+^) [37] compared with its adjacent kidney tissue (Fig. 1c). The expression of PGAM1 was suppressed by either rapamycin or ectopic expression of human *TSC2* cDNA in *Tsc2*-null Eker rat uterine leiomyoma cell line (ELT3) (Fig. 1d). Furthermore, both PGAM1 expression and mTOR activity were increased in human NSCLC tissues (Fig. 1e and Supplementary Figure S1). The results suggest that the expression of PGAM1 is mTOR dependent in rodent cells and human lung cancer.

**Fig. 2:**
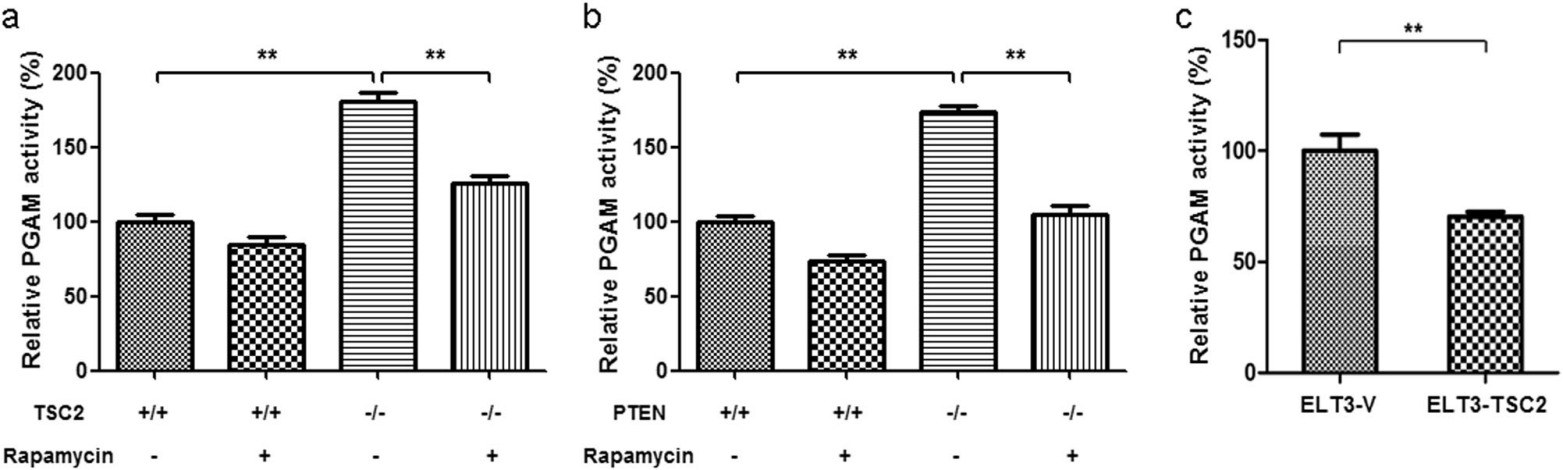
mTOR hyperactive cells have higher PGAM activity. Cell lysates extracted from WT, *Tsc2*^*−/−*^ **a** or *Pten*^*−/−*^ **b** MEFs treated with or without 10 nM rapamycin for 24 h were assayed for PGAM enzyme activity. **P < 0.001. **c** ELT3 cells with ectopic expression of TSC2 were subjected to enzyme activity measurement. **P = 0.002.Values represent the mean ± SD of triplicate samples

PGAM1 is a glycolytic enzyme catalyzing the transformation from 3-PG to 2-PG. To check PGAM enzymatic activity in cells with different mTOR state, we measured PGAM activity in total cell lysates. PGAM enzymatic activity was significantly increased in *Tsc2*^−/−^ and *Pten*^−/−^ MEF cells and was reduced by rapamycin treatment (Figs. 2a, b). Restoration of TSC2 in Tsc2 mutant ELT3 cells also reduced PGAM activity (Fig. 2c). mTOR-activated cells have more PGAM1 and therefore higher PGAM enzymatic activity.

### mTOR potentiates PGAM1 expression through up-regulation of HIF1α

**Fig. 3:**
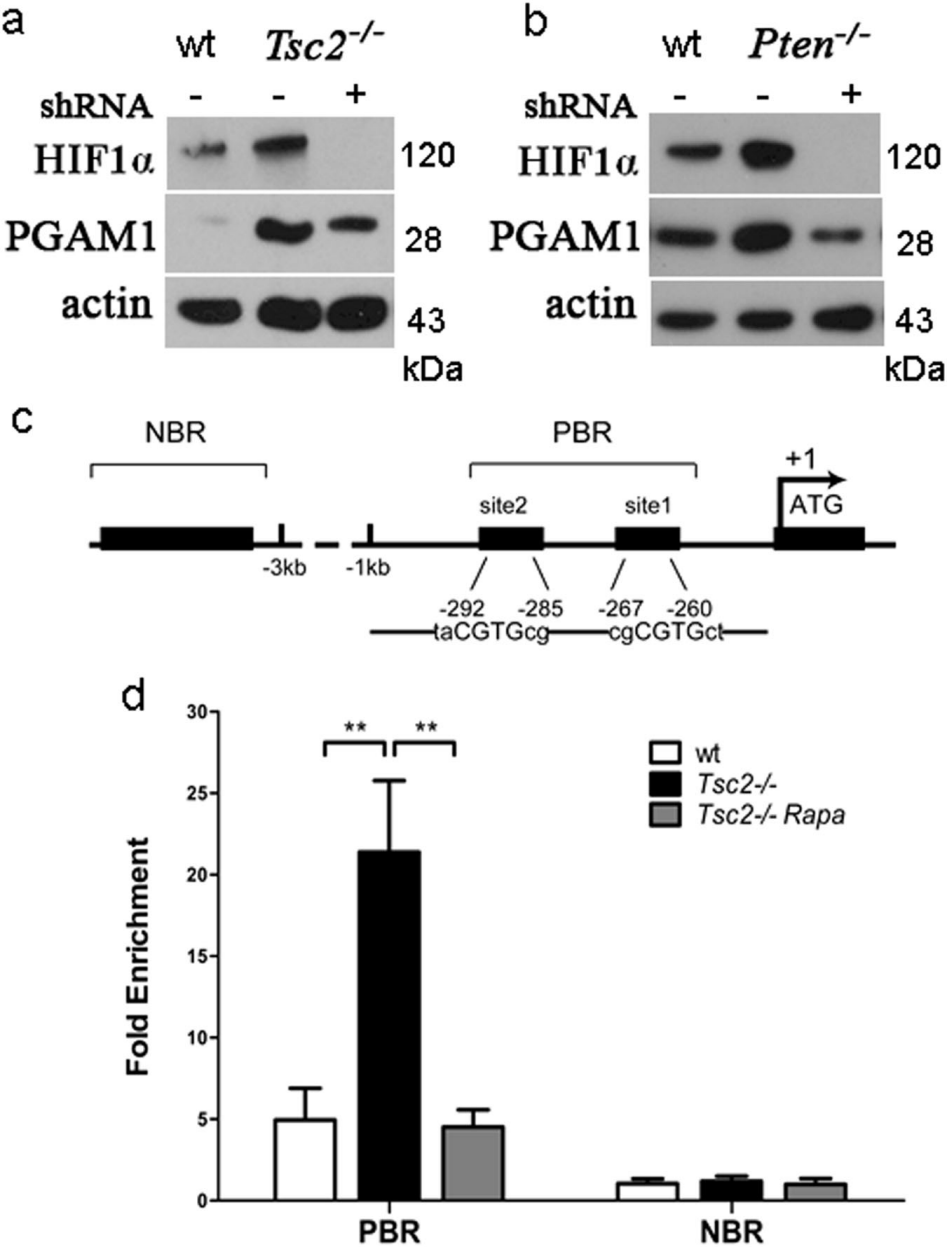
mTOR enhances PGAM1 expression through induction of HIF1α. Immunoblots of wt and *Tsc2*^*−/−*^ **a** or *Pten*^*−*^^*/−*^ **b** MEFs transduced with shHIF1α or scramble shRNA lentiviruses. **c** Schematic representation of the promoter regions of mouse PGAM1 gene. Site 1 and site 2 indicate the potential binding sequences of HIF1α on PGAM1 promoter. Predicted binding region (PBR) for real-time PCR covers both binding sites. The transcription start site is indicated by an arrow above the gene. **d** *Tsc2*^*−*^^/*−*^ MEFs was treated with or without 10 nM rapamycin (Rapa) for 24 h. HIF1α antibody-immunoprecipitated DNA from wt and *Tsc2*^*−*^^*/−*^ MEFs was PCR amplified for PBR and NBR regions. The data are plotted as the ratio of immunoprecipitated DNA subtracting nonspecific binding to IgG vs. total input DNA. Representative data from two independent experiments are shown. Data represent mean ± SEM of replicate real-time PCR. **P < 0.001

To dissect the mechanism of mTOR-augmented PGAM1 expression, we explored the role of an mTOR downstream target, HIF1α, which functions as a transcriptional activator in controlling cellular adaptation to hypoxia and glycolysis [38–40]. HIF1α expression was regulated by mTOR signaling and knockdown of HIF1α with two distinct short-hairpin RNAs (shRNAs) decreased PGAM1 expression in both *Tsc2*^−/^^−^ cells and *Pten*^−/−^ cells (Figs. 3a, b and Supplementary Figure S2), suggesting that mTOR stimulates PGAM1 expression through induction of HIF1α under normoxic condition. Next, we examined whether HIF1α stimulates PGAM1 expression at the transcription level by chromatin immunoprecipitation (ChIP) assay. Genomatix software analysis predicted that two HIF1α potential binding sites were next to each other in the promoter region of mouse *PGAM1* gene (Fig. 3c). Real-time PCR analysis of ChIP DNA revealed that HIF1α bound to a DNA region upstream of exon 1 in *PGAM1* gene and the binding affinity was significantly higher in *Tsc2*^−/^^−^ cells than in WT cells. Furthermore, the interaction between HIF1α and the PGAM1 promoter was disrupted by rapamycin treatment (Fig. 3d). We thus conclude that mTOR is a positive regulator of PGAM1 through HIF1α transcriptional activation.

### Blockage of PGAM1 suppresses oncogenic mTOR-mediated aerobic glycolysis, cell proliferation, and tumor development

**Fig. 4:**
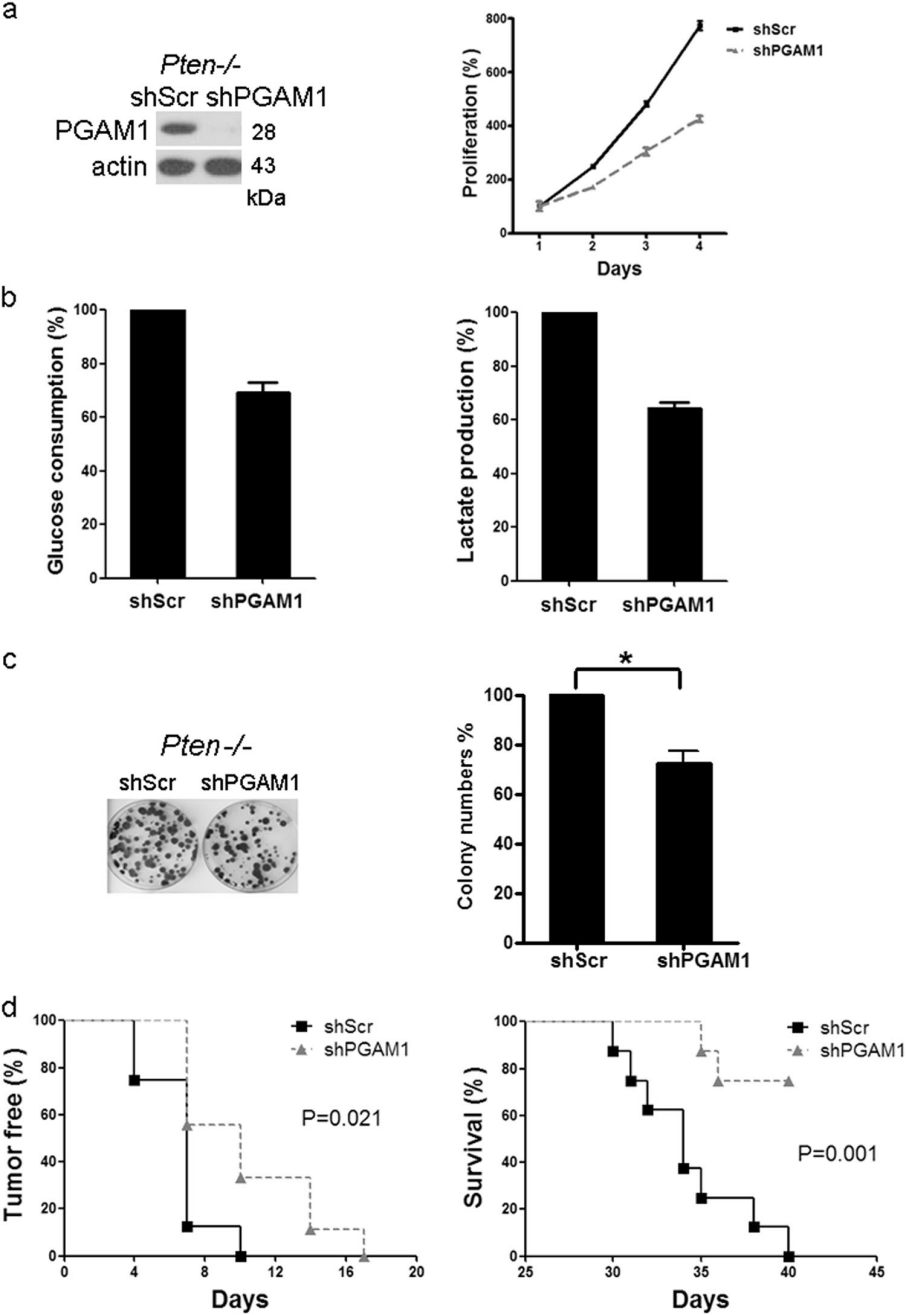
Knockdown of PGAM1 expression reduces proliferation, glycolysis, and tumor formation of mTOR hyperactive cells. **a** Left panel: Immunoblot of the *Pten*^*−/−*^ MEFs with or without shPGAM1 knockdown. (Right panel: The proliferation of PGAM1 knockdown cells and control cells was examined by the MTT assay. Values represent the mean ± SD of triplicate samples. *P* < 0.05. **b** The conditioned media from the cultures of *Pten*^*−*^^*/−*^ MEFs with or without shPGAM1 knockdown were examined for glucose consumption (left panel) and lactate production (right panel). Data represent mean ± SEM. *P* < 0.05. **c** Colony formation of PGAM1 knockdown cells. Values represent the mean ± SD of triplicate samples. *P < 0.05. **d** *Pten*^*−*^^*/−*^ MEFs transduced with shPGAM1 or scramble lentiviruses were inoculated subcutaneously into nude mice and monitored for tumor development (left) and survival (right). *P* < 0.05

As PGAM1 is critical for tumor growth [21] and is abundant in cells with hyperactive mTOR unveiled in this study, we checked whether the enhanced PGAM1 expression was essential for RTK-PI3K-AKT-mTOR-mediated aerobic glycolysis and oncogenesis. Two distinct shRNAs for PGAM1 were used to knock down PGAM1 in *Pten*^−/−^ MEFs and depletion of PGAM1 blocked the proliferation of *Pten*^−/−^ MEFs *in vitro* (Fig. 4a and Supplementary Figure S3a). Both glucose consumption and lactate production were decreased after PGAM1 knockdown (Fig. 4b and Supplementary Figure S3b), indicating elevated PGAM1 contributes to mTOR up-regulation of aerobic glycolysis. PGAM1-depleted *Pten*^−/−^ MEFs had reduced colony formation capacity (Fig. 4c). Furthermore, *Pten*^−/−^ MEFs expressing shPGAM1 or shScramble were subcutaneously injected into immunodeficient nude mice to determine whether PGAM1 depletion affects tumorigenesis. Reduction of PGAM1 significantly attenuated the tumor formation of *Pten*^−/−^ MEFs in nude mice and prolonged the survival of these tumor bearing mice (Fig. 4d and Supplementary Figure S3c). Taken together, PGAM1 plays an important role in mTOR-mediated aerobic glycolysis, cell proliferation, and tumorigenesis.

### PGAM1 expression correlates with mTOR activity in human NSCLC tissues and associates with patient poor prognosis

**Fig. 5:**
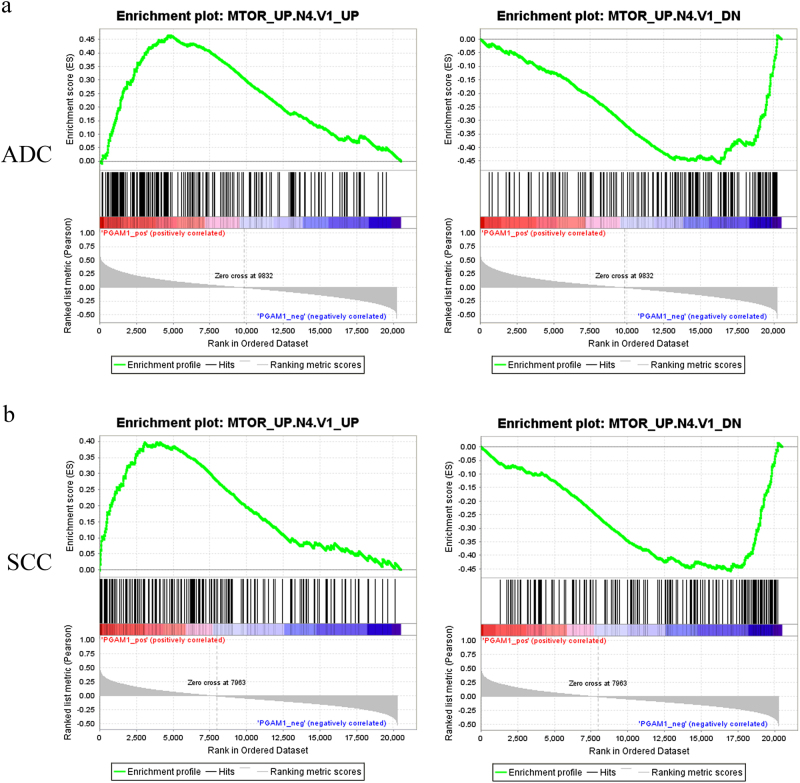
PGAM1 expression positively correlates with mTOR signaling pathway activity in human NSCLC. Based on PGAM1 expression levels, the gene set enrichment analyses were performed on NSCLC datasets from the TCGA database using the gene sets positively regulated by mTOR signaling (MTOR_UP.N4.V1_UP, the left panel) and the gene sets negatively regulated by mTOR signaling (MTOR_UP.N4.V1_DN, the right panel). **a** Analysis of 515 adenocarcinoma (ADC) patient samples. Left: Normalized Enrichment Score (NES) = 1.9564092, *P* < 0.01. Right: NES = −1.794588, *P* < 0.01. **b** Analysis of 501 squamous cell carcinoma (SCC) patient samples. Left: NES = 1.637388, *P* < 0.01. Right: NES = −1.8062531, *P* < 0.01. The barcode plot indicates the position of the genes in each gene set; red and blue colors represent positive and negative Pearson’s correlation with PGAM1 expression, respectively

To investigate the clinical relevance of this newly discovered mTOR regulation of PGAM1 in human cancer tissues, we analyzed The Cancer Genome Atlas (TCGA) RNAseq datasets for correlation between PGAM1 expression and mTOR signaling pathway activity in the tumors of 1016 NSCLC patients with Gene Set Enrichment Analysis (GSEA). The results showed that the positively regulated genes by mTOR signaling (gene set: MTOR_UP.N4.V1_UP, Fig. 5, left) were enriched in the PGAM1-high expression groups. Inversely, the negatively regulated genes by mTOR signaling (gene set: MTOR_UP.N4.V1_DN, Fig. 5, right) were enriched in the PGAM1-low expression groups, in both lung adenocarcinoma (ADC) (Fig. 5a) and lung squamous cell carcinoma (SCC) (Fig. 5b) subtypes of NSCLC. In contrast, the GSEA did not show the correlation between PGAM2 expression and mTOR signaling pathway activity in ADC tissues (Supplementary Figure S4a). In SCC tissues, the mTOR up-regulated signature (MTOR_UP.N4.V1_UP, the left panel) was correlated with PGAM2 low expression, and the correlation between the mTOR down-regulated signature (MTOR_UP.N4.V1_DN, the right panel) and PGAM2 high expression did not reach statistically significant (*P* = 0.06) (Supplementary Figure S4b). Therefore, PGAM1 expression positively correlates with mTOR activity in both ADC and SCC, and PGAM2 expression may inversely correlate with mTOR activity in SCC.

To validate the correlation we derived from the TCGA database, we checked PGAM1 or PGAM2 expression and mTOR activity in 227 NSCLC tissues along with patient clinicopathological characteristics. Immunohistochemical (IHC) staining was used to evaluate the levels of PGAM1 or PGAM2 expression and mTOR biomarker pS6. The clinicopathological features (age, gender, smoking status, histology, pathological T category, lymph node metastasis, distant metastasis, and clinical stage) of NSCLC patients are elaborated in Table 1. Tissue slices of 149 male and 78 female patients, with a mean age of 60.3 years (30–83 years), were used in IHC staining analysis. SCC, ADC, and large-cell lung cancer (LCLC) were included in this cohort study. The median survival time was 43 months, with follow-up time ranging from 1 to 120 months.

**Table 1:** Clinicopathologic features and analysis of PGAM1 and pS6 expression in tumor tissues of NSCLC patients

Characteristics	Total cases	PGAM1 staining		*P* value	pS6 staining		*P* value
		Low level (%)	High level (%)		Low level (%)	High level (%)	
Age (years)							
≤60	107	55 (51.4)	52 (48.6)	0.677	68 (63.5)	39 (35.4)	0.152
>60	120	65 (54.2)	55 (45.8)		65 (54.2)	55 (45.8)	
Gender							
Male	149	84 (56.4)	65 (43.6)	0.143	93 (62.4)	56 (37.6)	0.106
Female	78	36 (46.2)	42 (53.8)		40 (51.3)	38 (48.7)	
Smoking status							
Non smoker	77	34 (44.2)	43 (55.8)	0.060	39 (50.6)	38 (49.4)	0.082
Smoker	150	86 (57.3)	64 (42.7)		94 (62.7)	56 (37.3)	
Histology							
ADC	71	34 (47.9)	37 (52.1)	0.061	35 (49.3)	36 (50.7)	0.132
SCC	99	61 (61.6)	38 (38.4)		64 (64.6)	35 (35.4)	
LCLC	57	25 (43.9)	32 (56.1)		34 (59.6)	23 (40.4)	
Pathological T category							
pT1/pT2	182	102 (56.0)	80 (44.0)	0.054	110 (60.4)	72 (39.6)	0.255
pT3/pT4	45	18 (40.0)	27 (60.0)		23 (51.1)	22 (48.9)	
Lymph node metastasis							
Absent	122	72(59.0)	50 (41.0)	0.045	76 (62.3)	46 (37.7)	0.222
Present	105	48 (45.7)	57 (54.3)		57 (54.3)	48 (45.7)	
Distant metastasis							
Absent	205	117 (57.1)	88 (42.9)	<0.001	128 (62.4)	77 (37.6)	<0.001
Present	22	3 (13.6)	19 (86.4)		5 (22.7)	17 (77.3)	
Clinical stage							
Stage I/II	127	80 (63.5)	47 (36.5)	0.001	81 (63.8)	46 (36.2)	0.074
Stage III/IV	100	40 (40.0)	60 (60.0)		52 (52.0)	48 (48.0)	
pS6 expression							
Low level	133	102 (76.7)	31 (23.3)	<0.001			
High level	94	18 (19.1)	76 (80.9)				

*χ*^2^ Test. *ADC* adenocarcinoma, *NSCLC* non-small cell lung cancer, *PGAM1* phosphoglyceric acid mutase-1, *pS6* protein S6, *SCC* squamous carcinoma, *LCLC* large-cell lung cancer

**Fig. 6:**
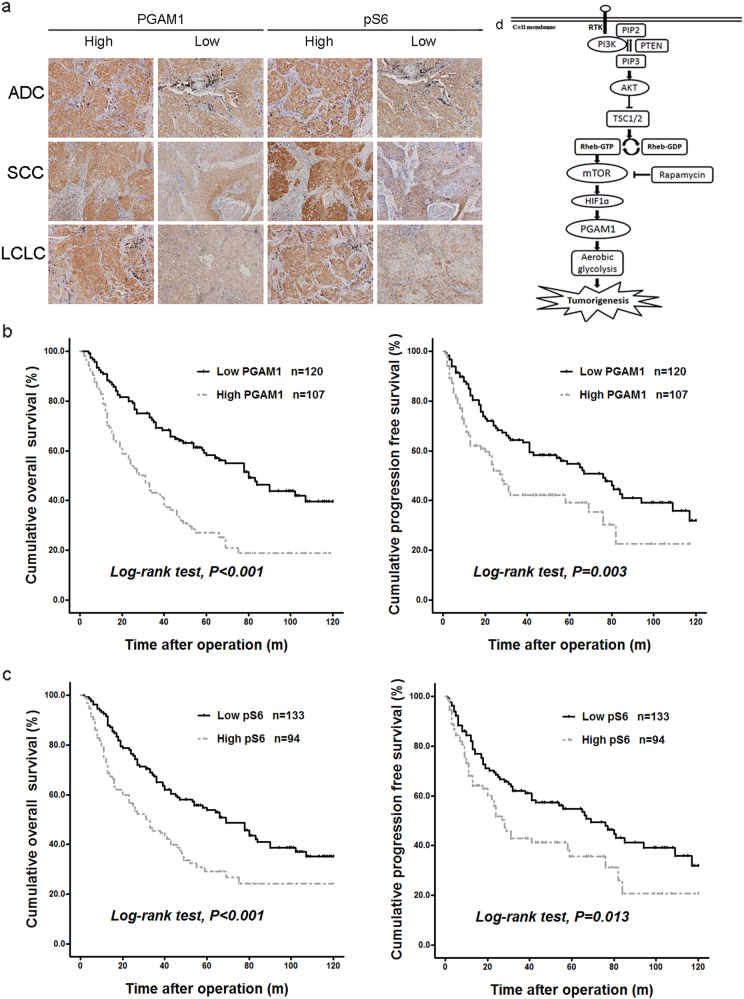
PGAM1 expression correlates with mTOR signaling in tumor tissues and patient prognosis of human NSCLC. **a** IHC analysis of 227 paraffin-embedded human NSCLC tumors including adenocarcinomas (ADCs), squamous cell carcinomas (SCCs), and large-cell lung cancer (LCLC) (× 200) for the abundance of PGAM1 and pS6 (Ser235/236). Representative pictures of high and low expression were shown. **b**, **c** Kaplan–Meier survival curves illustrate the overall survival (OS, left) and progression-free survival (PFS, right) of NSCLC patients in respect to the expressions of PGAM1 **b** and pS6 **c**. **d** Schematic illustration of mTOR regulation of PGAM1, aerobic glycolysis, and tumorigenesis

High PGAM1 expression of cancer tissues was observed in 47.1% of the patients (107 of 227), while high pS6 level was observed in 41.4% of the patients (94 of 227) (Fig. 6a). The expression pattern of PGAM1 and pS6 was analyzed by *χ*^2^ test (Table 1). High PGAM1 expression was significantly associated with lymph node metastasis (*P* = 0.045), distant metastasis (*P* < 0.001, and clinical stage (*P* = 0.001). As for age, sex, smoking status, histology, and T stage, no significant association was observed between the high- and low-PGAM1 groups. High pS6 level was significantly associated with distant metastasis (*P* < 0.001), but not with other clinicopathological parameters (*P* > 0.05). High PGAM1 expression significantly correlated with high pS6 abundance (*P* < 0.001) (Table 1).

Next, we evaluated the relationship between the abundance of PGAM1 and pS6 and NSCLC patient prognosis. Subjects with a high PGAM1 expression had much shorter median overall survival (OS) and progression-free survival (PFS) time than those with a low PGAM1 expression (28 vs. 80 months and 28 vs. 76 months, respectively). Subjects with a high pS6 had much shorter median OS and PFS time than those with a low pS6 (31 vs. 69 months and 28 vs. 69 months, respectively). Kaplan–Meier survival analyses further indicated high PGAM1 expression or increased pS6 was associated with poor prognosis of NSCLC patients (Figs. 6b, c). High PGAM1 was associated with significantly shorter OS and PFS (log-rank, *P* < 0.001, *P* = 0.003, respectively). High pS6 also associated with worse OS and PFS (log-rank, *P* < 0.001, *P* = 0.013, respectively).

The staining pattern of PGAM2 was different from that of PGAM1 (Fig. 6a vs. Supplementary Figure S5a). High PGAM2 in cancer tissues was observed in 9.3% of the patients (Supplementary Table S1). And high- and low-PGAM2 groups were not associated with aforementioned parameters and mTOR activity (Supplementary Table S1) (*P* > 0.05). PGAM2 expression is not associated with prognosis of NSCLC patients (log-rank, *P* = 0.975 for OS, *P* = 0.458 for PFS, respectively) (Supplementary Figure S5b).

## Discussion

Our study demonstrates that mTOR signaling pathway activates PGAM1 through up-regulation of HIF1α. Depletion of PGAM1 suppressed glycolysis and tumorigenesis caused by oncogenic mTOR signaling. Augmented PGAM1 expression and enhanced mTOR activity positively correlated in NSCLC tissues. Elevated PGAM1 conferred these patients with poor prognosis (Fig. 6d).

mTOR is a master regulator of cell metabolism, survival, growth, proliferation, and differentiation [8–10,41]. As a downstream effector for many frequently mutated oncogenic pathways, aberrant activation of mTOR often exists in tumors [42]. We have found previously that mTOR signaling is a positive regulator of the Warburg effect, through up-regulation of PKM2 and some other glycolytic enzymes [11]. Overexpressed PGAM1 has been observed in multiple cancers [21–26]. In this study, we identified mTOR as a stimulator of PGAM1 expression in cell lines, Tsc2 mutant mouse kidney tumor sample and human NSCLC tissues. mTOR enhancement of PGAM1 expression is independent of p53 as PGAM1 was elevated in *Tsc2*^*−/−*^ MEFs and sensitive to rapamycin treatment even though p53 was absent in both *Tsc2*-deficient cells and their control MEFs [35]. In consistent with their increased abundance of PGAM1, mTOR-active cells also have higher PGAM enzymatic activity.

mTOR activates HIF1α through increasing its expression [11,43–46]. HIF1α is a transcriptional activator of several glycolytic enzymes that coordinates cell metabolism and growth [14]. Our study identified HIF1α as a key mediator in mTOR activation of PGAM1 expression. Reduction of HIF1α decreased PGAM1 expression in mTOR hyperactivated *Tsc2*^−/−^ and *Pten*^−/−^ cells. Binding of HIF1α on the promoter of *PGAM1* gene was stronger in *Tsc2*^−/−^ cells than in WT cells, and was disrupted by rapamycin treatment. mTOR is thus an activator of PGAM1 expression through HIF1α-mediated transcriptional activation.

NSCLC is the leading cause of tumor-related death worldwide. Around 74% squamous cell lung cancer samples harbor alterations in RTK/PI3K/AKT/mTOR pathway (RTK 26% and PI3K 47%) [47]. Cancers may show high mTOR pathway activity without an associated genetic or genomic alteration of canonical RTK/PI3K/AKT/mTOR pathway [42, 47, 48]. Furthermore, mTOR activity is not only influenced by genomic alterations but also by epigenomic alterations of these signaling cascades [42]. To seek clinical relevance of mTOR-PGAM1 signaling cascade in human cancer, we analyzed TCGA NSCLC RNAseq datasets with GSEA and our NSCLC tumor tissues with IHC staining, respectively. In contrast to PGAM2, PGAM1 expression well correlated with mTOR signaling in NSCLC. Elevated pS6 and PGAM1 conferred NSCLC patients with poor prognosis.

Our previous study have identified that mTOR is a crucial regulator of aerobic glycolysis, cell growth, and proliferation [11, 12]. The findings from current study demonstrate that PGAM1 plays critical roles in mTOR-mediated Warburg effect and tumor growth. Disruption of PGAM1 decreased glycolysis in mTOR-activated cells. Furthermore, reduction of PGAM1 in these cells suppressed cell proliferation *in vitro* and blunted tumor formation of these cells in nude mice. Therefore, we suggest that PGAM1 is a downstream effector of mTOR signaling and a potential target for cancer treatment. PGMI-004A, a small molecular inhibitor of PGAM1, was found to decrease glycolysis, PPP flux, and biosynthesis, and consequently block cell proliferation and tumor growth [21]. In addition, Evans et al. [49] identified small-molecule MJE3 could covalently bind to PGAM1 and suppressed breast cancer cell proliferation. Thus, inhibition of PGAM1 is a promising strategy that targets both glycolysis and biosynthetic pathways for the treatment of cancer [21, 50].

In summary, mTOR is a positive regulator of PGAM1. As a downstream effector of mTOR, PGAM1 is critical for oncogenic mTOR-mediated cell proliferation and tumor formation. mTOR activation of PGAM1 renders NSCLC patients with reduced survival. Our studies serve as a proof of concept that the components in this cascade may be targeted for the treatment of diseases caused by abnormal RTK/PI3K/AKT/mTOR signaling pathway.

## Materials and methods

### Reagents

Rapamycin was purchased from Sigma-Aldrich; Dulbecco’s modified Eagle’s medium (DMEM), fetal bovine serum, 4–12% Bis-Tris Nu-PAGE gels and Lipofectamine 2000 were from Invitrogen. All restriction enzymes and SYBR Green Supermix were from Takara.

### Antibodies

The antibodies against pS6 (Ser235/236) and S6 for immunoblotting have been described previously [34]. TSC2 and β-actin antibodies and all the HRP-labeled secondary antibodies were from Santa Cruz Biotechnology. HIF1α antibody for immunoblotting was from Novus Biologicals and for ChIP was from Abcam. PGAM1 antibody for immunoblotting was from Abcam and for IHC was from Novus Biologicals. pS6 antibody for IHC was from R&D Systems. PGAM2 antibody for IHC was from Abcam.

### Cell culture

All MEF cells have been described previously [11, 29, 41]. ELT3 cells were transduced with control retroviruses or retroviruses carrying TSC2 cDNA to generate ELT3-V and ELT3-TSC2 cells [51, 52].

### Immunoblotting assay

Whole cells were lysed in lysis buffer (2% sodium dodecyl sulfate, 10% glycerol, 10 mM Tris, pH 6.8, 100 mM dithiothreitol), boiled for 10 min, and then subjected to immunoblotting later as described previously [34].

### Mouse kidney tumor for immunoblotting

Kidney tumor and adjacent kidney tissue from heterozygous Tsc2 exon 3 deletion (*Tsc2*^del3/+^) mouse were sonicated and extracted for immunoblotting using lysis buffer [29].

### Human NSCLC tumor for immunoblotting

Human NSCLC cancer tissues and their adjacent tissues were dissected at the Tianjin Medical University Cancer Institute and Hospital (Tianjin, China). Tissue samples were sonicated and extracted for immunoblotting using the lysis buffer described above.

### RNA extraction and quantitative real-time PCR analysis

Total RNA was isolated with TRIzol according to the manufacturer’s instructions. Thermocycling condition is stage 1, 10 s at 95 °C; stage 2, 40 cycles, with 1 cycle consisting of 5 s at 95 °C, 34 s at 60 °C; stage 3, dissociation stage. The relative expression level of PGAM1 mRNA for each sample was calculated as 2^−ΔΔCt^ (sample).

The primers used are as follows: PGAM1 forward, AGCGACACTATGGCGGTCT and PGAM1 reverse, TGGGACATCATAAGATCGTCTCC; actin forward, AGAGGGAAATCGTGCGTGAC and actin reverse, CAATAGTGATGACCTGGCCGT.

### PGAM enzymatic activity assay

The activity of PGAM was determined by enzyme-coupled assay according to previous publications [20, 53]. Cell lysates for PGAM enzymatic activity were prepared in NP-40 lysis buffer (with phenylmethylsulfonyl fluoride 1:100). One hundred microliters of the reaction mixture solution (100 mM Tris-HCl, pH 8.0, 1 mM EDTA, 4 mM MgCl_2_, 200 mM KCl, 0.4 mM NADH, 3 mM ADP, 20 μM 2,3-bisphosphoglycerate, lactate dehydrogenase (1.2 unit/ml), pyruvate kinase (1 unit/ml), enolase (0.6 unit/ml)) was put to each well in 96-well plate and each sample with 20 μg total protein (total volume of 50 µl with lysis buffer) were then added. After incubation at 37 °C for 15 min, 50 µl solution that contained 4 mM 3-PG and 100 mM Tris-HCl (pH 8.0) was loaded to each well. PGAM activity was measured by the decrease of absorbance at 340 nm in the linear range of velocity, which was defined as Δ*A*/min (slope of the curve).

### Knockdown of PGAM1 and HIF1α in MEF cells

The shRNAs were used to target mouse HIF1α and PGAM1. For HIF1α, the lentiviral plasmid was purchased from Dharmacon and the target sequence is: AACTGGAAATCATCATCCA. For HIF1α-2, PGAM1, and PGAM1-2, the forward and reverse primers were designed and synthesized by Takara. These primers were annealed and cloned into pLL3.7 vector. The target sequences are as follows: HIF1α-2, GCAGACCCAGTTACAGAAACC; PGAM1, GGGCATCCCTATCGTCTATGA, and PGAM1-2, GCAAAGCCATGGAAGCTGT.

For HIF1α, pLKO.1-shRNA and packaging plasmid (psPAX2, pMD2.G) were cotransfected into 293 T cells. For HIF1α-2, PGAM1, and PGAM1-2, lentiviruses were generated by cotransfecting pLL3.7-shRNA and the packaging vectors (VSVG, REV, and pMDL) into 293 T cells. Forty-eight hours after transfection, viruses were collected and used for MEF cell transduction.

### ChIP assay

Details for ChIP assay were described previously [11]. Briefly, cells were fixed with 1% (vol/vol) formaldehyde, and the reaction was then stopped by the addition of 0.125 M glycine. The cells were lysed by lysis buffer and were incubated on ice for 10 min. The lysates were then sonicated and spun down. Salmon Sperm DNA/Protein A agarose 50% slurry (Millipore) were used to preclear the supernants for 5 h at 4 °C. One hundred microliters of each sample were removed and stored (total input). The rest of the samples were then immunoprecipitated with polyclonal anti-HIF1α antibody or rabbit control IgG overnight at 4 °C. The immunocomplexes were captured with 40 µl of Salmon Sperm DNA/Protein A agarose for 1 h with rotation at 4 °C. The immune complexes/beads were then collected and washed, and the antibody/histone/DNA complexes were extracted twice with the elution buffer. The eluates were pooled, and the cross-linking was reversed with the addition of 0.2 M NaCl and incubation at 65 °C overnight. The DNA was purified and amplified by real-time PCR. The relative amount of the targeted sequences precipitated by the antibody was calculated relative to IgG control and normalized to total input samples using the following equation: fold enrichment = 2^(−ΔΔCt [ChIP/NIS]). Ct [ChIP/NIS] = (Ct [ChIP]−(Ct [Input]−Log 2 (Input Dilution Factor)))−(Ct [IgG]−(Ct [Input]−Log 2 (Input Dilution Factor))).

The primers indicated are as follows: PBR-F, TCATCCCTAAAGAGGAAGCGC and PBR-R, AAGTTCTGCGGCCTGCTAAG; NBR-F, GGACATAGGATACCTCAGGCACA and NBR-R, GCGAAAGGACAAAGGGAACT.

### Measurements of glucose and lactate

Glucose consumption and lactate production have been described previously [11]. Briefly, 6–8 × 10^4^ cells per well were seeded in 12-well plates for 1 day, and then changed to fresh culture medium for 2 days. The medium was collected and the glucose and lactate levels were examined immediately using EKF-C-LINE glucose and lactate analyzer (EKF Diagnostics). The glucose consumption and lactate production were normalized to cell numbers.

### Cell proliferation assay (MTT) and colony formation assay

Cells were seeded in 96-well plates and incubated with 100 μl of fresh medium containing 20 μl MTT reagent (5 mg/ml in PBS) at 37°C for 1–4 h. The spectrometric absorbance at 490 nm was determined and the viability ratio was calculated. For colony formation, cells were seeded in 10-cm tissue culture dishes for up to 2 weeks and assay was stopped when the colonies were visible with naked eyes. Colonies were stained with crystal violet for analysis.

### Xenografting tumorigenesis

Subcutaneous tumors were established as described previously [34]. Immunodeficient nude mice (strain BALB/c, 6–8 weeks old) were obtained from the Institute of Laboratory Animal Sciences, Chinese Academy of Medical Sciences (CAMS), China. Eight mice (4 males and 4 females) were used in each cohort. Tumor growth and mouse survival were assessed over 2-month periods following subcutaneous inoculation of 1 × 10^6^ *Pten*^*−*^^/*−*^ cells with shRNA vector or shPGAM1/shPGAM1-2 in 0.1 ml DMEM into the right posterior dorsum. Animal protocols were approved by the Animal Center of the Institute of basic Medical Sciences, CAMS and PUMC and were compliant with the regulation of Beijing Administration Office of Laboratory Animal on the care of experimental animals. The Kaplan–Meier log-rank test was used for analysis of mouse survival with GraphPad Prism software.

### Gene set enrichment analysis

GSEA is a computational method that determines whether a priori defined set of genes shows statistically significant, concordant differences between two biological states [54]. We examined the enrichment of both mTOR signaling positively and negatively regulated gene signatures in PGAM1 high and low expressing NSCLC tissues using the GSEA analytic software provided by the Broad Institute (http://www.broadinstitute.org/gsea/index.jsp). The gene sets used in this study were MTOR_UP.N4.V1_UP and MTOR_UP.N4.V1_DN from C6 oncogenic signatures of the Molecular Signatures Database (MSigDB) v.6.1 (http://software.broadinstitute.org/gsea/msigdb/index.jsp). RNA sequencing data of NSCLC including 501 cases of lung SCC and 515 cases of lung ADC were obtained from TCGA datasets (http://cancergenome.nih.gov/). Parameters used in the current study were as follows: 1000 random sample permutations were used to calculate the *P* value. *P* value < 0.05 was considered as significant.

### Patients and specimens

PGAM1 or PGAM2 expression and mTOR status were evaluated in dissected tumor tissues from 227 (149 male and 78 female) patients with NSCLC who underwent surgery without prior chemical or radiation therapy at Tianjin Medical University Cancer Institute and Hospital between January 2004 and December 2011. The patients ranged in age from 30 to 83 years (mean, 60.3 years). Clinical information of each case was obtained from the medical records. All patients were classified according to Union for International Cancer Control (UICC)/TNM classification, the 7th edition. Follow-up data after surgery were available for all patients with a median follow-up period of 43 months (range, 1–120 months). This study was approved by the Research Ethics Committee of Tianjin Medical University Cancer Institute and Hospital.

### Tissue microarray

Tissue microarrays (TMAs) were constructed as described [55]. All samples were reviewed histologically with hematoxylin and eosin staining. The TMA specimens were manufactured by Shanghai Outdo Biotech (Shanghai, China).

### Immunohistochemistry

Specimens were deparaffinized and rehydrated. The sections were incubated overnight at 4 °C with primary antibody, incubated 1 h at room temperature with second antibody, and then visualized with diaminobenzidine. All immunoreactions were evaluated independently by two pathologists without knowledge of patients’ clinical records. Staining was scored as follows: intensity (0 = negative, 1 = weak, 2 = moderate, 3 = strong), and percentage of positive tumor cells (1 = 1–50%, 2 = 51–75%, 3 = > 76%), the scores of each sample were multiplied to give a final score. Subjects with score > 6 were defined as high expression, while subjects ≤ 6 were defined as low expression.

### Statistical analysis

Statistical analysis of group differences was done using the *χ*^2^ test. The Kaplan–Meier method was used for survival analysis and differences in survival were estimated using the log-rank test. All statistical analyses were done with the IBM SPSS Statistics software (version 21; IBM, Armonk, NY, USA).

## Electronic supplementary material

Supplementary Table 1

Supplementary Figure 1

Supplementary Figure 2

Supplementary Figure 3

Supplementary Figure 4

Supplementary Figure 5

Supplementary Figure Legends
