# Supplementary material for: Phosphoglyceric acid mutase-1 contributes to oncogenic mTOR-mediated tumor growth and confers non-small cell lung cancer patients with poor prognosis
Source: Cell Death Differ. 2018 Jan 23;25(6):1160–73. doi: 10.1038/s41418-017-0034-y (PMC5988759; doi:10.1038/s41418-017-0034-y)
Supplement: Supplementary file 1 — Supplementary Table 1 [file 41418_2017_34_MOESM1_ESM.docx]

**Supplementary Table S1** Clinicopathologic features and analysis of PGAM2 expression in tumor tissues of Non-Small Cell Lung Cancer patients

| Characteristics | Total cases | **PGAM2 staining** | | p value |
| --- | --- | --- | --- | --- |
|  |  | Low level (%) | High level (%) |  |
| **Age(year)** |  |  |  | 0.680 |
| ≤60 | 107 | 98(91.6) | 9(8.4) |  |
| >60 | 120 | 108 (90) | 12(10) |  |
| **Gender** |  |  |  | 0.068 |
| Male | 149 | 139(93.3) | 10(6.7) |  |
| Female | 78 | 67(85.9) | 11(14.1) |  |
| **Smoking status** |  |  |  | 0.952 |
| Non smoker | 77 | 70(90.9) | 7(9.1) |  |
| Smoker | 150 | 136(90.7) | 14(9.3) |  |
| **Histology** |  |  |  | 0.121 |
| ADC | 71 | 61(85.9) | 10(14.1) |  |
| SCC | 99 | 90(90.9) | 9(9.1) |  |
| LCLC | 57 | 55(96.5) | 2(3.5) |  |
| **Pathological T**  **category** |  |  |  | 0.214 |
| pT1/pT2 | 182 | 163(89.6) | 19(10.4) |  |
| pT3/pT4 | 45 | 43(95.6) | 2(4.4) |  |
| **Lymph node**  **metastasis** |  |  |  | 0.294 |
| Absent | 122 | 113(92.6) | 9(7.4) |  |
| Present | 105 | 93(88.6) | 12(11.4) |  |
| **Distant**  **metastasis** |  |  |  | 0.978 |
| Absent | 205 | 186(90.7) | 19(9.3) |  |
| Present | 22 | 20(90.9) | 2(9.1) |  |
| **Clinical stage** |  |  |  | 0.205 |
| Stage I/II | 127 | 118(92.9) | 9(7.1) |  |
| Stage III/IV | 100 | 88(88.0) | 12(12.0) |  |
| **pS6 expression** |  |  |  |  |
| Low level | 133 | 120(90.2) | 13(9.8) | 0.746 |
| High level | 94 | 86(91.5) | 8(8.5) |  |

Chi-square test; ADC, adenocarcinoma; SCC, squamous carcinoma; LCLC, large cell

lung cancer.
