# Supplementary figures and images for: Phosphoglyceric acid mutase-1 contributes to oncogenic mTOR-mediated tumor growth and confers non-small cell lung cancer patients with poor prognosis
Source: Cell Death Differ. 2018 Jan 23;25(6):1160–73. doi: 10.1038/s41418-017-0034-y (PMC5988759; doi:10.1038/s41418-017-0034-y)

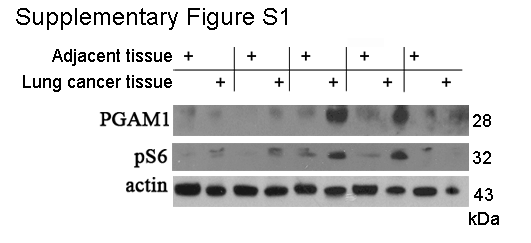

Supplement: Supplementary file 2 — Supplementary Figure 1 [file 41418_2017_34_MOESM2_ESM.tif]

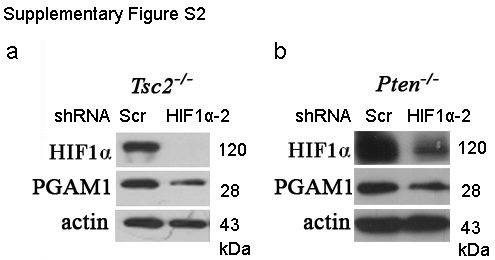

Supplement: Supplementary file 3 — Supplementary Figure 2 [file 41418_2017_34_MOESM3_ESM.tif]

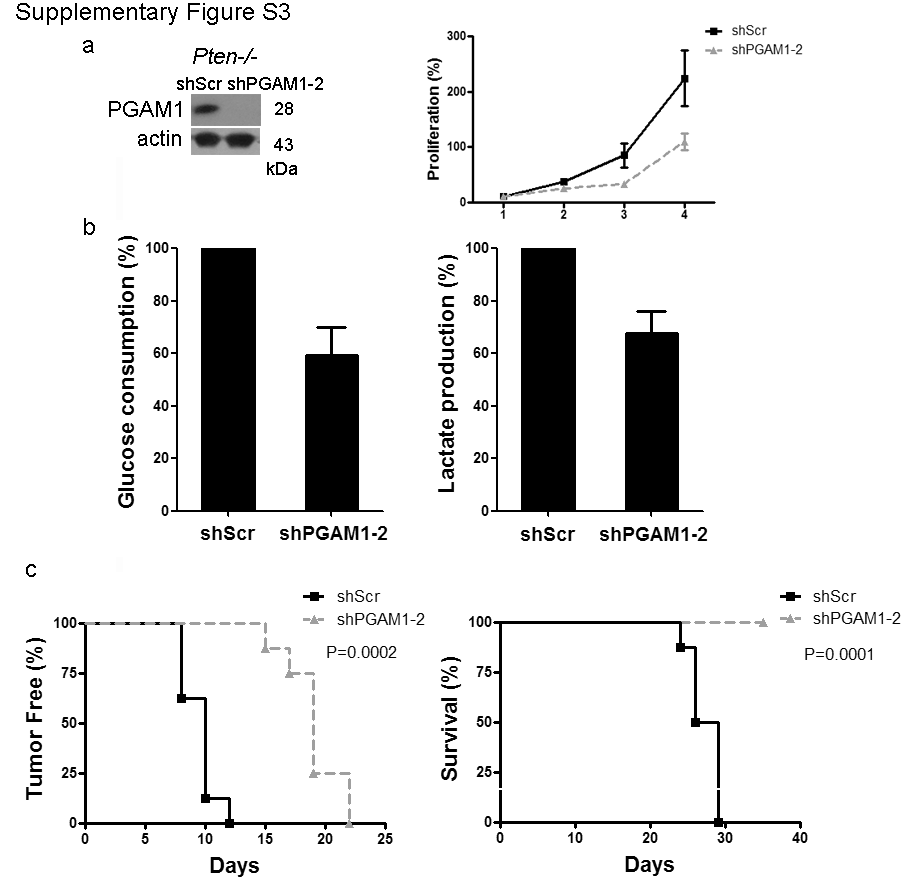

Supplement: Supplementary file 4 — Supplementary Figure 3 [file 41418_2017_34_MOESM4_ESM.tif]

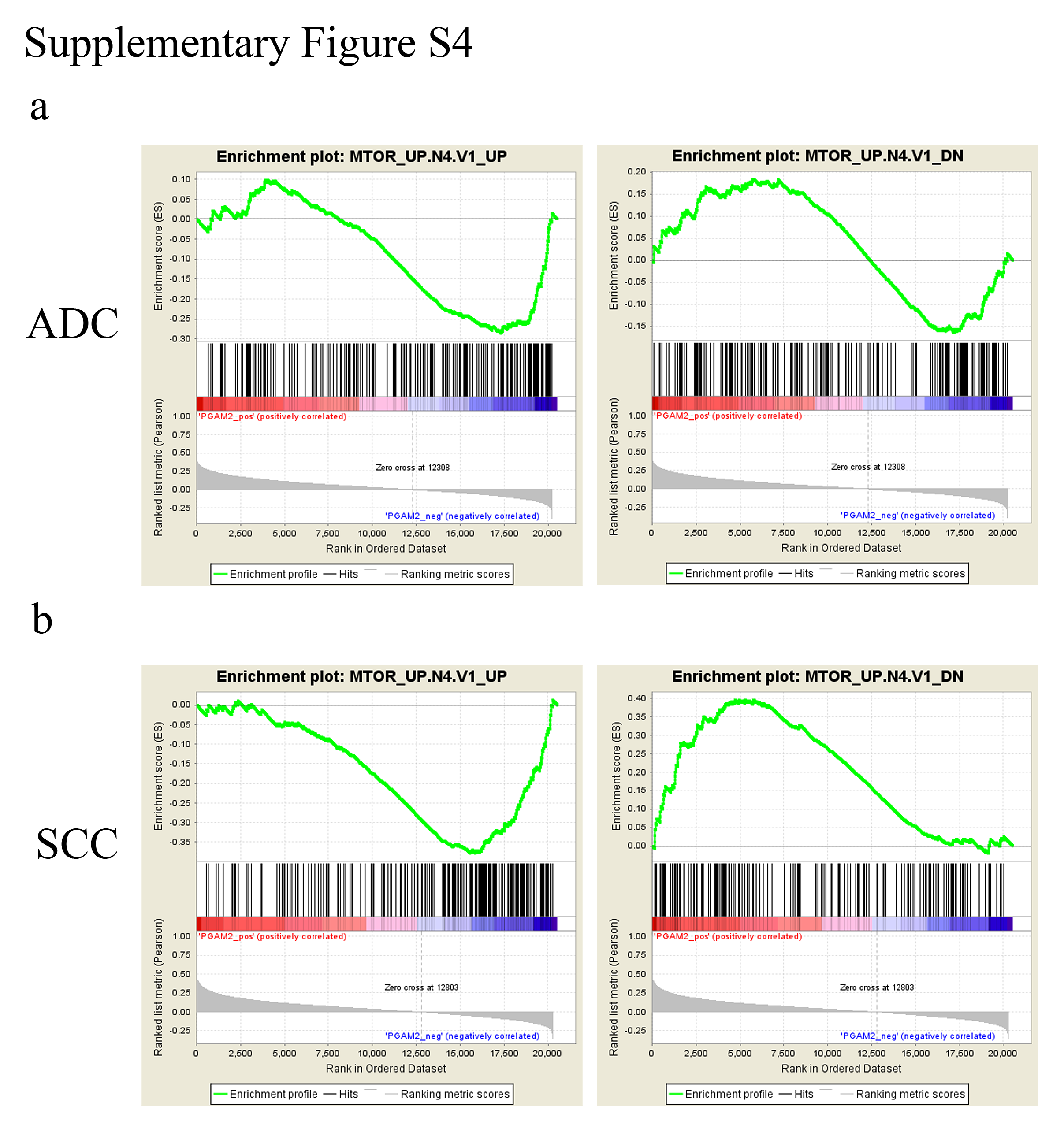

Supplement: Supplementary file 5 — Supplementary Figure 4 [file 41418_2017_34_MOESM5_ESM.tif]

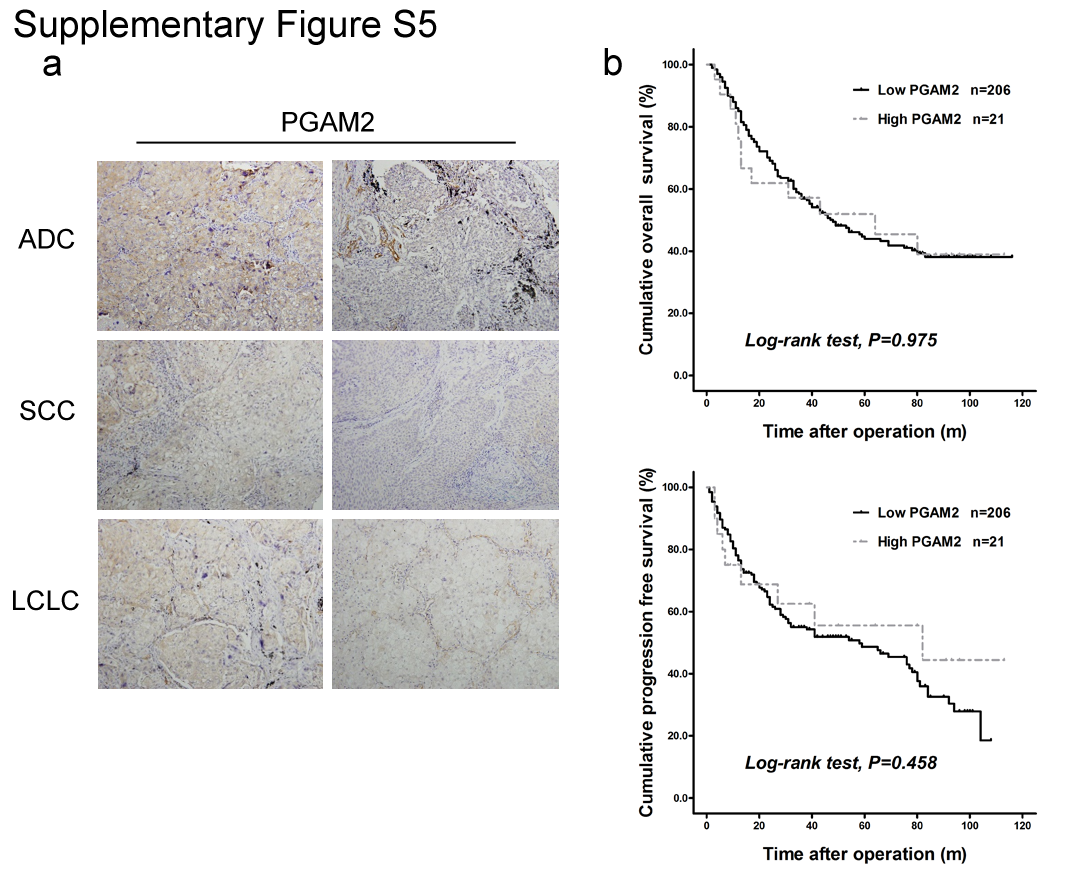

Supplement: Supplementary file 6 — Supplementary Figure 5 [file 41418_2017_34_MOESM6_ESM.tif]
