# Supplementary material for: Phosphoglyceric acid mutase-1 contributes to oncogenic mTOR-mediated tumor growth and confers non-small cell lung cancer patients with poor prognosis
Source: Cell Death Differ. 2018 Jan 23;25(6):1160–73. doi: 10.1038/s41418-017-0034-y (PMC5988759; doi:10.1038/s41418-017-0034-y)
Supplement: Supplementary file 7 — Supplementary Figure Legends [file 41418_2017_34_MOESM7_ESM.docx]

**Supplementary Figure Legends**

**Supplementary Figure S1** PGAM1 expression and mTOR activation in NSCLC. Protein lysates were extracted from five pairs of human NSCLC tissues and adjacent tissues and then subjected to immunoblotting.

**Supplementary Figure S2** Knockdown of HIF1α suppresses PGAM1 expression. Immunoblots of *Tsc2^-/-^* (a) and *Pten^-/-^* (b) MEFs transduced with shHIF1α-2 or scramble shRNA lentiviruses.

**Supplementary Figure S3** Knockdown of PGAM1 reduces proliferation, glycolysis and tumor formation of mTOR hyperactive cells. (a) Left panel: Immunoblot of *Pten^-/-^* MEFs with or without shPGAM1-2 knockdown. Right panel: The proliferation of PGAM1-2 knockdown cells and control cells was examined by MTT assay. Values represent the mean ± SD of triplicate samples. P < 0.05. (b) The conditioned media from the cultures of *Pten^-/-^* MEFs with or without shPGAM1-2 knockdown were examined for glucose consumption (Left) and lactate production (Right). Data represent mean ± SEM. P < 0.05. (c) *Pten^-/-^* MEFs transduced with shPGAM1-2 or scramble lentiviruses were inoculated subcutaneously into nude mice and monitored for tumor development (left) and survival (right), P < 0.05.

**Supplementary Figure S4** PGAM2 expression does not positively correlates with mTOR signaling pathway activity in human NSCLC. Based on PGAM2 expression levels, the gene set enrichment analyses were performed on NSCLC datasets from TCGA database using the gene sets positively regulated by mTOR signaling (MTOR_UP.N4.V1_UP, the left panel) and the gene sets negatively regulated by mTOR signaling (MTOR_UP.N4.V1_DN, the right panel). (a) Analysis of 515 adenocarcinoma (ADC) patient samples. Left: Normalized Enrichment Score (NES)= -1.2091627, P= 0.20. Right: NES= 0.6930516, P=0.86. (b) Analysis of 501 squamous cell carcinoma (SCC) patient samples. Left: NES=-1.607811, P=0.02. Right: NES= 1.5154966, P= 0.06. The barcode plot indicates the position of the genes in each gene set; red and blue colors represent positive and negative Pearson’s correlation with PGAM2 expression, respectively.

**Supplementary Figure S5** PGAM2 expression does not correlate with mTOR signaling and patient prognosis of human NSCLC. (a) IHC analysis of 227 paraffin-embedded human NSCLC tumors including adenocarcinomas (ADC), squamous cell carcinomas (SCC) and large cell lung cancer (LCLC) (×200) for the abundance of PGAM2. Each picture was captured from the same microarray that as shown in Figure 6a. (b) Kaplan–Meier survival curves illustrate the overall survival (OS, upper) and progression free survival (PFS, lower) of NSCLC patients in respect to the expressions of PGAM2.
